# Efficacy of the Mannheim Peritonitis Index (MPI) in Predicting Postoperative Outcomes in Patients With Perforation Peritonitis

**DOI:** 10.7759/cureus.83193

**Published:** 2025-04-29

**Authors:** Sparsh Gupta, Anand Zingade, Mayur Baviskar, Riya B Vakil

**Affiliations:** 1 General Surgery, Pimpri Chinchwad Municipal Corporation's Postgraduate Institute, Yashwantrao Chavan Memorial Hospital, Pune, IND

**Keywords:** intestinal perforation, mannheim peritonitis index, mpi, perforation peritonitis, peritonitis

## Abstract

Introduction: Peritonitis or inflammation of the peritoneum is a leading cause of mortality in surgical patients. Perforation of the gastrointestinal tract (perforation peritonitis) is one of the commonest origins and a rapidly fatal disease. The Mannheim Peritonitis Index (MPI) is a scoring system used to forecast outcomes for individuals suffering from peritonitis that uses a straightforward bedside grading method. This study aimed to determine how well the MPI predicts clinical outcomes for individuals who have perforation peritonitis in an Indian setting.

Methodology: This study was a prospective observational study conducted over two years at a tertiary care hospital in India with a sample size of 72 patients. The study population comprised patients aged over 18 years who presented to the emergency department with clinical and radiological suspicion of perforation peritonitis, which was later confirmed intraoperatively. Pregnant women and patients below 18 years of age were excluded. The variables found in the MPI were assigned the values indicated, and the total MPI score for each patient was calculated. The cases were then grouped into three groups, viz those with an MPI below 21 points, between 21 and 29 points, and those above 29 points. Patients were followed up, and outcomes were recorded in terms of discharge, morbidity or mortality. Morbidity was specifically measured in terms of surgical site infection, wound dehiscence, intra-abdominal abscess formation, acute renal failure and pulmonary complications.

Results: Higher MPI scores on presentation directly correlated with a greater severity of disease in terms of post-operative morbidity and mortality. The overall mortality rate was 16.7%, with a clear correlation between higher MPI scores and increased mortality. As far as post-operative systemic complications were concerned, patients with a higher preoperative MPI were more likely to develop complications like acute kidney injury and pulmonary complications like pneumonia postoperatively. Surgical site infections occurred in 44.4% of patients, and wound dehiscence in 16.7%. Of the four patients who subsequently developed an intra-abdominal abscess, three had an MPI greater than 29 on presentation.

Conclusion: The findings of this study demonstrate that the MPI is a valuable tool for stratifying patients of perforation peritonitis, with higher MPI scores correlating with worse clinical outcomes, including organ failure, wound complications, and mortality. The demographic analysis of our study revealed a significant male predominance and a relatively young patient population, suggesting regional or demographic factors. The high incidence of organ failure, particularly among those with higher MPI scores, underscores the critical nature of perforation peritonitis and the necessity for intensive care support.

## Introduction

Peritonitis is inflammation of the peritoneum. It may be classified as either primary or secondary, localised or generalised, or based on the presence of infectious agents (septic or non-septic). Secondary peritonitis is mostly septic and often results from gastrointestinal tract pollution. It can be secondary to trauma, ischemic damage, neoplasia, ulceration, foreign body blockage, or dehiscence of previous surgical wounds. Gastrointestinal contents, including bacteria and digestive enzymes, seep into the peritoneal cavity following perforation of a hollow viscus. This sets off an inflammatory reaction resulting in peritonitis, sepsis, and circulatory collapse.

Depending on where the perforation occurred, perforation peritonitis might appear differently clinically. For instance, odynophagia, vomiting, and abrupt chest pain are common symptoms of oesophageal perforations, but sudden, severe stomach pain is usually the first sign of gastroduodenal perforations. Conversely, colonic perforations could proceed more slowly and result in localised abscesses or secondary bacterial peritonitis. Abdominal examination findings include tenderness, guarding, and systemic inflammatory symptoms such as hypotension and tachycardia. Perforation peritonitis is a surgical emergency, and the objectives of surgery are to identify and treat the infection's cause, lower the abdomen's infectious burden, and facilitate healing.

One of the commonest causes is perforation of the gastrointestinal tract, which causes extensive inflammation and infection of the peritoneal cavity (perforation peritonitis) that can be rapidly fatal. With its quick development, it poses a serious issue, with mortality ranging from 10% to 40%, even with recent advances in medical and surgical therapy [1].

Accurate risk classification systems help better predict patient outcomes, prioritise resources, and customise treatment plans. Numerous scores, such as the Sequential Organ Failure Assessment (SOFA) score, the Mannheim Peritonitis Index (MPI), and the Acute Physiology and Chronic Health Evaluation (APACHE) score, have been created for this purpose. Although the APACHE score is considered one of the most effective scoring systems for assessment of disease severity, it is not specific to peritonitis. Factors such as the aetiology of the perforation or type of peritonitis are not included, potentially making MPI a more effective tool in this clinical setting.

The MPI was first described in Germany in the 1980s to forecast outcomes for individuals suffering from peritonitis, using a straightforward grading method that considered clinical, laboratory and operative findings [2]. Age, gender, organ failure, malignancy, preoperative length of peritonitis, source of sepsis, extent of peritonitis, and the type of exudate are the eight prognostic criteria included. The overall score may vary from 0 to 47, with a particular value allocated to each element. According to a pilot study by Billing et al., higher MPI scores are associated with more severe illness and a greater probability of death [3]. While MPI has shown prognostic value in prior studies, its utility in Indian populations, characterised by unique demographic and clinical features, remains underexplored. Furthermore, in resource-limited settings like India, bedside prognostic tools play an important role in prognosticating patients.

## Materials and methods

This study was a prospective observational study conducted over two years (June 2022 to December 2024) in the Department of General Surgery at a tertiary care hospital in Western Maharashtra, India. The sample size was calculated using the following formula:

\[ n = \frac{\text{DEFF} \times N \times p(1 - p)}{\left( \frac{a^2}{Z^2_{1 - \alpha/2}} \times (N - 1) \right) + p(1 - p)} \]

Where hypothesised percentage frequency of the outcome factor in the population (p): 14% ±10; confidence limits as a percentage of 100 (absolute ±%): 10%; Z is the value from the standard normal distribution corresponding to the desired confidence level (Z = 2.68 for 95% CI); p is the expected true proportion = 3.3% (p = 0.045), hence q = 1-p = 0.955; e is the desired precision = 5% (e = 0.05).

Adding these values to the above formula results in a sample size (n) of 47 [4]. If we add 10% missing data or non-response, the minimum required sample size will be 52. Our study had a final sample size of 72 patients who fit the inclusion criteria.

The study population comprised patients aged over 18 years who presented to the emergency department with clinical and radiological suspicion of perforation peritonitis, later confirmed intraoperatively. Pregnant women and patients below 18 years of age were excluded. The study protocol was approved by the institutional ethics committee, and written informed consent was obtained from all participants, with adherence to the standards for research involving human participants.

On presentation, using history, clinical examination, laboratory investigations, and intraoperative findings, variables found in the MPI were assigned the values indicated (Table 1), and the total MPI score for each patient was calculated by adding these. The minimum possible score was zero if no adverse factors were present, and the maximum was 47 if the presence of all was confirmed. Organ failure included renal, cardiovascular, pulmonary and gastrointestinal insufficiency. Oliguria (<20 mL/hour), elevated creatinine (>1.6 mg/dL), or elevated urea (>60 mg/dL) was counted as acute kidney injury. Low pO_2_ (<50 mmHg) or high pCO_2_ (>50 mmHg) represented respiratory insufficiency, while the presence of shock represented cardiovascular failure. Intestinal obstruction was defined as either bowel paralysis lasting more than 24 hours or a complete mechanical obstruction.

**Table 1:** Mannheim Peritonitis Index Source: Reference [2]

Risk Factor	Adverse	Points Assigned	Favourable	Points Assigned
Age	>50 years	5	<50 years	0
Gender	Female	5	Male	0
Malignancy	Present	4	Absent	0
Organ dysfunction	Present	7	Absent	0
Evolution time	>24 hours	4	<24 hours	0
Origin	Non colonic	4	Colonic	0
Extent of peritonitis	Generalised	6	Localised	0
Peritoneal exudate	Fecal	12	Clear	0
	Purulent	6		

After diagnosis, every patient underwent the required operative procedure at the earliest. Choice of incision and most appropriate procedure (e.g., resection and anastomosis, primary closure, stoma creation, etc.) and other operative decisions were left to the lead surgeon on a case-by-case basis. Adequate wash of at least three to four litres of warm saline was ensured in every case. Postoperative care was provided as necessary.

Demographic personal data such as age and gender, dates of admission and discharge from the hospital, days hospitalised, date of surgery and information related to the surgical procedure performed and operative findings were recorded. Patients were followed up for 30 days postoperatively, and outcomes were recorded in terms of discharge, morbidity, or mortality. Morbidity was further specifically measured in terms of postoperative surgical site infections, wound dehiscence, intra-abdominal abscess formation, acute renal failure and pulmonary complications. A surgical site infection was defined as an infection occurring up to 30 days after surgery near the surgical site. Acute renal failure was defined by the RIFLE criteria (Risk, Injury, Failure, Loss of kidney function, and End-stage kidney disease) [5]. Pulmonary complications were identified by pulmonary insufficiency, which was defined as the presence of any of the following on clinical examination, arterial blood gas analysis or chest X-ray. This included an oxygen saturation of less than 85% on room air, or pO_2_ < 50 mmHg or pCO_2_ > 50 mmHg on arterial blood gas analysis. Chest X-ray findings included the presence of radiological signs like bat-wing opacities, Kerley B lines, upper lobe diversion, peribronchial cuffing and airspace opacification.

Cases were divided into three groups based on their total MPI score, similar to the papers by Billing et al. [3], Notash et al. [6], and Ntirenganya et al. [7]. These included those with a total score below 21 points, those between 21 and 29 points, and those above 29 points, for further analysis and prognostication. The correlation between total MPI score and patient morbidity and mortality was analysed to determine if the MPI is an effective prognostic index in patients with perforation peritonitis. Statistical analysis was performed using IBM SPSS Statistics for Windows, Version 26 (Released 2020; IBM Corp., Armonk, New York, United States).

## Results

This prospective observational study was carried out at a tertiary care teaching institute in Pune, India, over two years, with a study population of 72 patients. Our relevant findings in terms of age and sex distribution, mortality rates, and postoperative morbidity in terms of surgical site infections, wound dehiscence, intra-abdominal abscess formation, and renal and pulmonary complications are shown below.

Age and gender distribution

**Figure 1:**
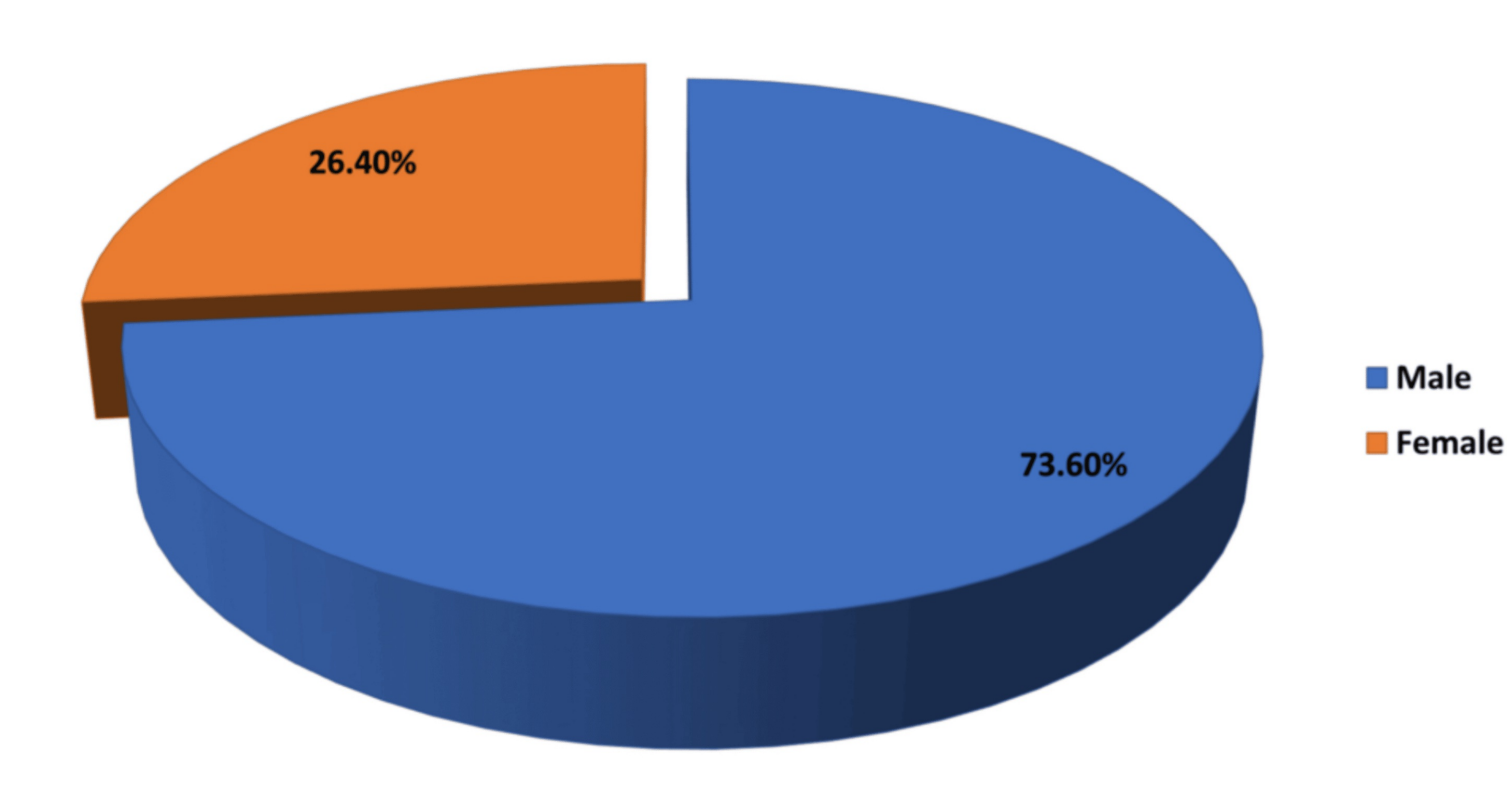
Gender Distribution of the Study Population

The majority of our patients lie in the 20 to 50 year age bracket, with a strong male predominance. The age and gender distribution of our population was given in Table 2 and Figure 1.

**Table 2:** Age Distribution of Study Population

Age Distribution	Number of Subjects	Percentage of Total
≤20 yrs	6	8.3
21-30 yrs	16	22.2
31-40 yrs	15	20.8
41-50 yrs	15	20.8
51-60 yrs	6	8.3
>60 yrs	14	19.4
Total	72	100

Source of perforation

**Figure 2:**
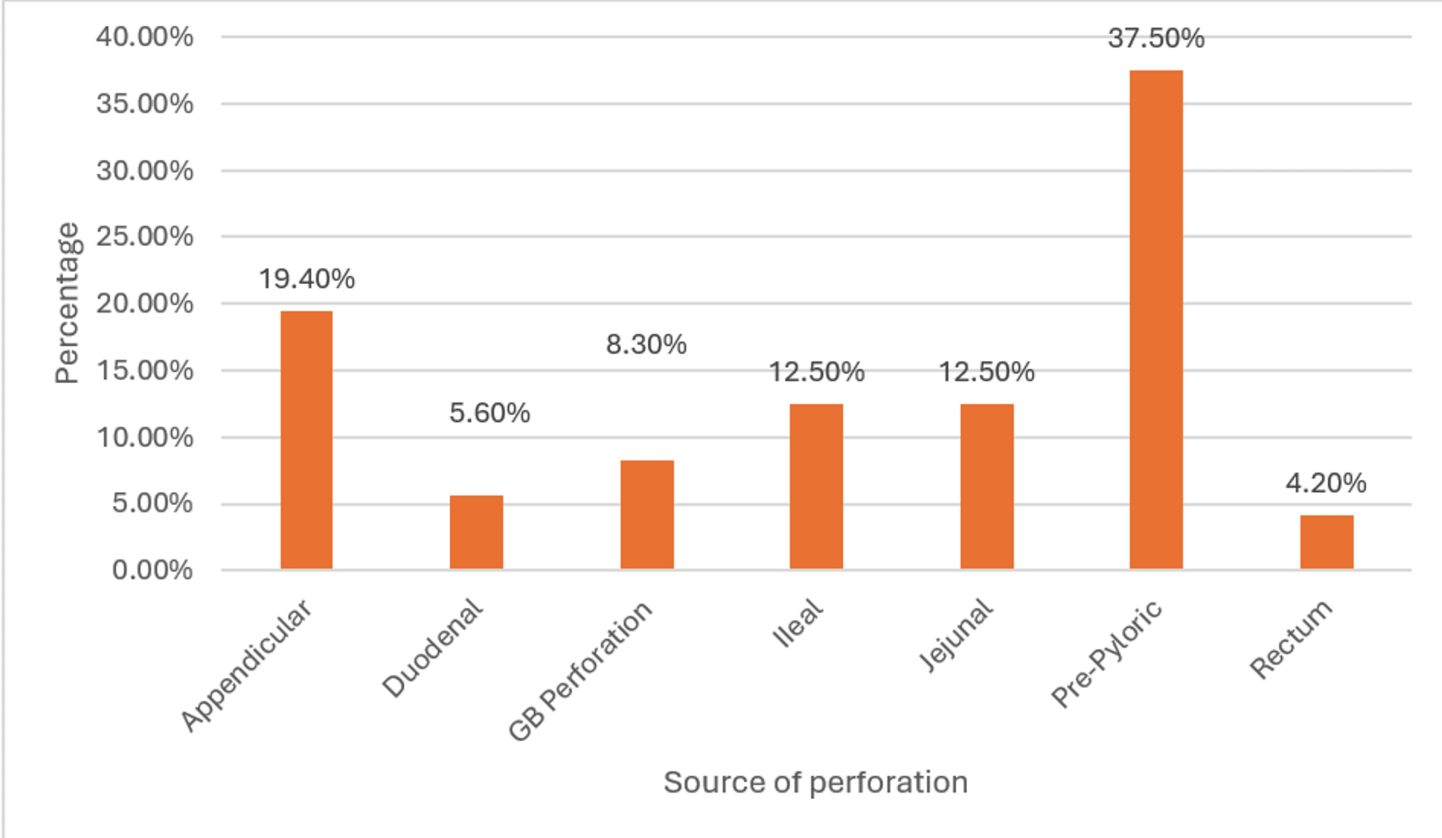
Source of Perforation GB: Gallbladder

An analysis of the source of perforation found intra-operatively showed the leading cause to be pre-pyloric in the study. This likely correlates with the lower socioeconomic strata that our hospital primarily serves, as well as the unique demographic characteristics of the area (Table 3 and Figure 2).

**Table 3:** Source of Perforation

Source of Perforation	Number of Patients	Percentage
Appendicular	14	19.4
Duodenal	4	5.6
Gallbladder	6	8.3
IIeal	9	12.5
Jejunal	9	12.5
Pre-pyloric	27	37.5
Rectum	3	4.2
Total	72	100

Mortality

**Figure 3:**
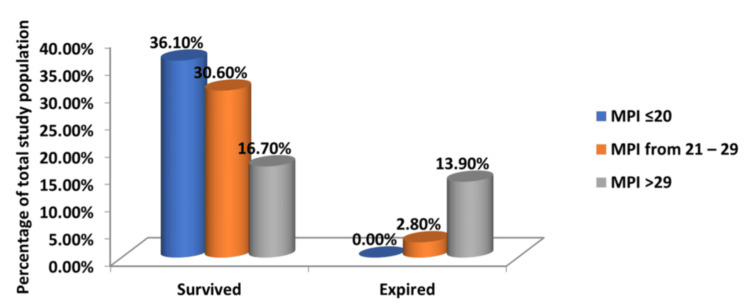
Relationship Between Mortality and MPI Scores in the Study Population MPI: Mannheim Peritonitis Index

Of the study population of 72 patients, 60 survived, while 12 succumbed. Of the 12 patients that succumbed, 10 had an MPI score greater than 29, while almost half the patients that survived (26 out of 60) had an MPI score less than 20 on admission. A chi-square test was performed to interpret the results, and the p-value was found to be <0.05. This indicates a statistically significant association, highlighting the strong prognostic value of the score in terms of postoperative mortality. The relationship between MPI and mortality rates is present in Table 4 and Figure 3.

**Table 4:** Relationship Between Mortality and MPI Scores in the Study Population MPI: Mannheim Peritonitis Index

Mortality	Group 1	Group 2	Group 3	Total
	MPI <21	MPI 21-29	MPI >29	
Survived	26	22	12	60
Expired	0	2	10	12
Total	26	24	22	72
% of Total	0	2.80	13.90	16.70

Postoperative complications: surgical site infection, wound dehiscence and intra-abdominal abscess formation

**Figure 4:**
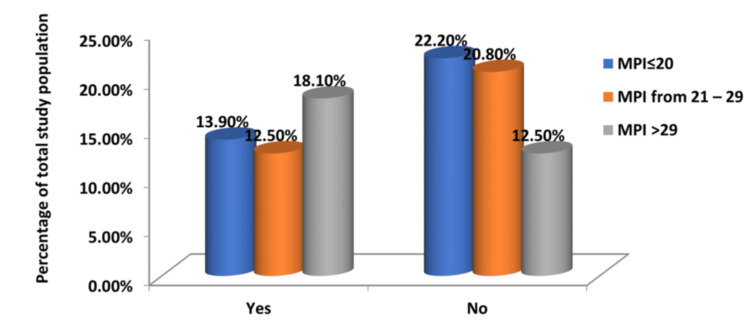
Relationship of MPI Scores and Development of Postoperative Surgical Site Infection MPI: Mannheim Peritonitis Index

**Figure 5:**
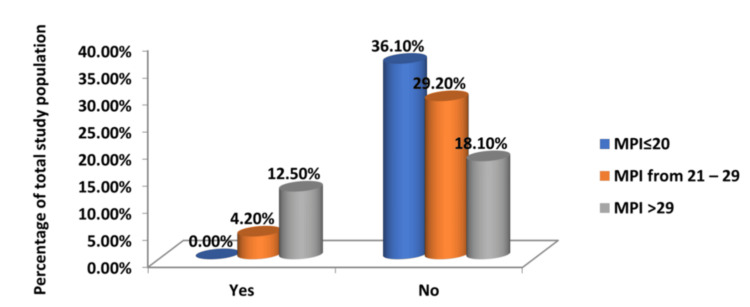
Relationship Between MPI Score and Postoperative Wound Dehiscence MPI: Mannheim Peritonitis Index

**Figure 6:**
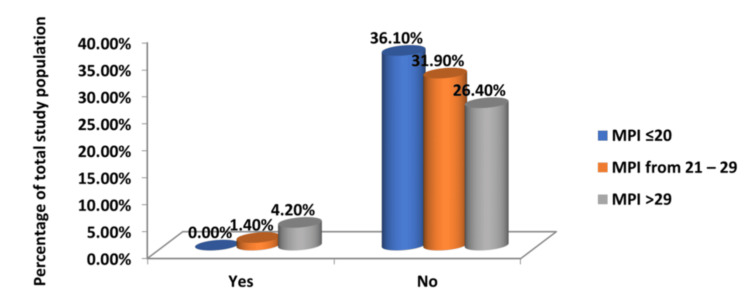
Relationship of MPI Score and Development of Intra-abdominal Abscess in the Postoperative Period MPI: Mannheim Peritonitis Index

The postoperative morbidity of the patients who survived was analysed. Postoperative complications were present in a significant proportion of patients. Surgical site infections occurred in 44.4% of patients, and wound dehiscence was found in 16.7%. Of the four patients who subsequently developed an intra-abdominal abscess, three had an MPI greater than 29 on presentation (Tables 5-7 and Figures 4-6).

**Table 5:** Relationship of MPI Scores and Development of Postoperative Surgical Site Infection MPI: Mannheim Peritonitis Index

Surgical Site Infection	Group 1	Group 2	Group 3	Total
	MPI <21	MPI 21-29	MPI >29	
Yes	10	9	13	32
No	16	15	9	40
Total	26	24	22	72
% of Total	14	12.50	18.10	44.40

**Table 6:** Relationship Between MPI Score and Postoperative Wound Dehiscence MPI: Mannheim Peritonitis Index

Wound Dehiscence	Group 1	Group 2	Group 3	Total
	MPI <21	MPI 21-29	MPI >29	
Yes	0	3	9	12
No	26	21	13	60
Total	26	24	22	72
% of Total	0	4.20	12.50	16.70

**Table 7:** Relationship of MPI Score and Development of Intra-abdominal Abscess in the Postoperative Period MPI: Mannheim Peritonitis Index

Intra-abdominal Abscess	Group 1	Group 2	Group 3	Total
	MPI <21	MPI 21-29	MPI >29	
Yes	0	1	3	4
No	26	23	19	68
Total	26	24	22	72
% of Total	0	1.40	4.20	5.60

Postoperative renal and pulmonary complications

**Figure 7:**
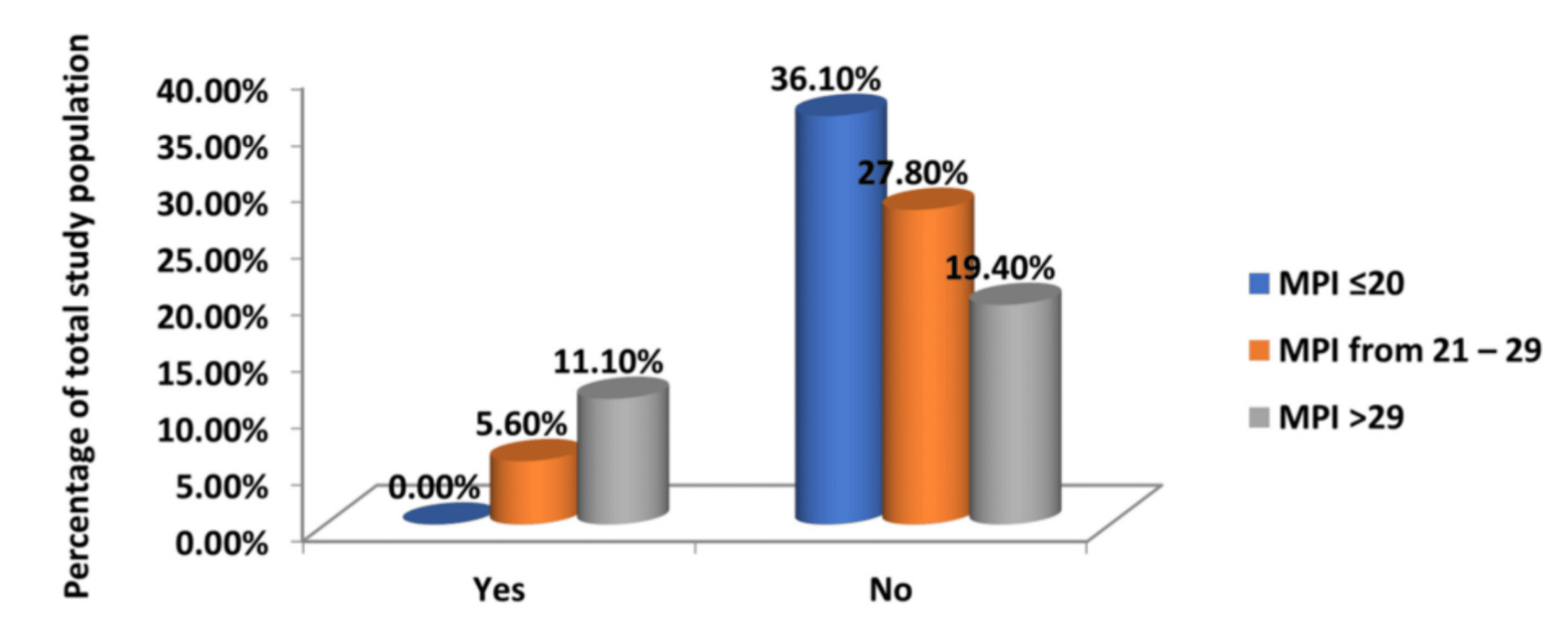
Relationship of MPI Score and Development of Acute Kidney Injury in the Postoperative Period MPI: Mannheim Peritonitis Index

**Figure 8:**
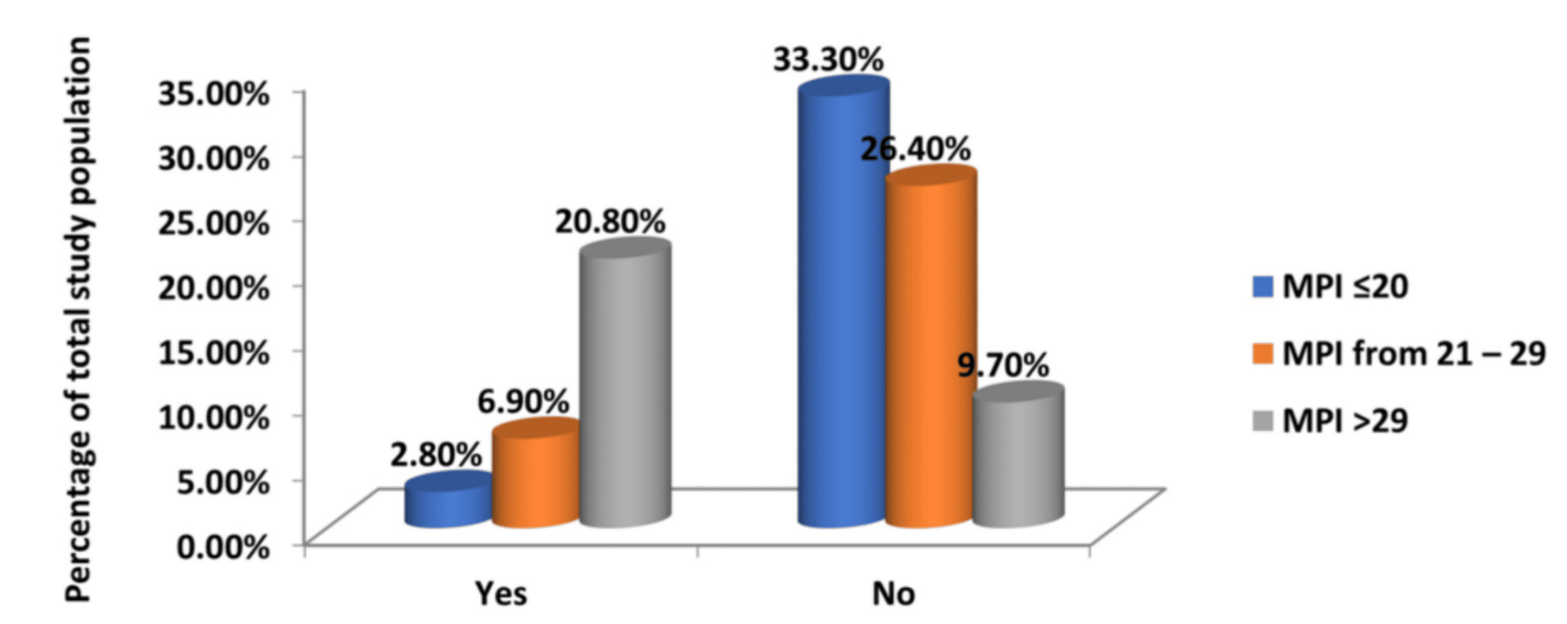
Relationship Between MPI Scores and Development of Pulmonary Complications in the Postoperative Period MPI: Mannheim Peritonitis Index

Higher MPI scores also directly correlated with a higher incidence of renal and pulmonary complications, reinforcing the predictive value of the MPI and validating the index as a useful tool for anticipating surgical risks (Tables 8, 9 and Figures 7, 8). The chi-square tests for each of these yielded a statistically significant result (p < 0.05), thereby establishing a significant association and underscoring the score's robust prognostic value in predicting postoperative complications.

**Table 8:** Relationship of MPI Score and Development of Acute Kidney Injury in the Postoperative Period MPI: Mannheim Peritonitis Index

Acute Kidney Injury	Group 1	Group 2	Group 3	Total
	MPI <21	MPI 21-29	MPI >29	
Yes	0	4	8	12
No	26	20	14	60
Total	26	24	22	72
% of Total	0	5.60	11.10	16.70

**Table 9:** Relationship Between MPI Scores and Development of Pulmonary Complications in the Postoperative Period MPI: Mannheim Peritonitis Index

Pulmonary Complications	Group 1	Group 2	Group 3	Total
	MPI <21	MPI 21-29	MPI >29	
Yes	2	5	15	22
No	24	19	7	50
Total	26	24	22	72
% of Total	2.8`	6.90	20.80	30.60

## Discussion

The MPI score ranges from 0 to 47, with higher scores indicating greater severity and higher risk of mortality in perforation peritonitis. This study's MPI scores ranged from 10 to 42, with a mean of 24.95 ± 7.91. Higher MPI scores were associated with worse outcomes in terms of mortality and morbidity.

The inflammation of the peritoneal cavity is known as peritonitis, and it is a leading cause of mortality in surgical patients. Peritonitis may be classified as either primary or secondary, localised or generalised, or based on the presence of infectious agents (septic or non-septic). Secondary peritonitis is commonly due to gastrointestinal leakage into the peritoneal cavity, which may be caused by trauma, ischemic damage, neoplasia, ulceration, foreign body blockage, or dehiscence of previous surgical wounds.

The prognosis of peritonitis remains very poor despite developments in diagnosis and management [8-11]. Early identification of patients with severe peritonitis and grading its severity may help in selecting patients for an aggressive surgical approach. Categorising patients into different risk groups would help prognosticate the outcome, select patients for intensive care and determine operative risk, thereby helping to choose the nature of the operative procedure, e.g. damage control versus definitive procedure, requirement for intensive care facilities, etc. Various scoring systems have been used to assess the prognosis and outcome of peritonitis. The present study was undertaken to evaluate the efficacy of the MPI scoring system in predicting individual prognostic outcomes in patients with perforation peritonitis in an Indian setting.

The MPI was developed by Wacha and Linder in 1983, based on the retrospective analysis of data from 1253 patients with peritonitis, in which 20 possible risk factors were considered. Of these, only eight proved to be of prognostic relevance and were entered into the MPI [2].

In our study, demographic analysis revealed that the mean age of participants was 41.98 ± 16.53 years, with ages ranging from 18 to 76 years. A detailed analysis of the age distribution showed that a significant portion of patients fell within the 21-30 (22.2%) and 31-40 (20.8%) age groups. This contrasts with findings from Liu et al. [12], who reported a higher prevalence of peritonitis in older age groups. The younger age distribution in the current study may reflect specific regional or environmental factors that influence the incidence of perforation peritonitis, such as dietary habits, prevalence of gastrointestinal infections, and access to healthcare.

However, younger age groups often presented with lower MPI scores, suggesting less severe disease and better prognosis, which is consistent with the findings of Liu et al. [13], who reported that younger patients tend to have better outcomes in perforation peritonitis due to fewer comorbidities and better overall health.

The gender distribution showed a significant male predominance (73.6%), which is in line with findings from Mishra et al. [14], who also observed a higher incidence of peritonitis among males. This male predominance may be attributed to higher rates of risk factors such as smoking, alcohol consumption, and occupational hazards in males, as well as potential differences in healthcare-seeking behaviour.

The overall mortality rate was 16.7%, with a clear correlation between higher MPI scores and increased mortality. This correlation aligns with the findings of Pathak et al. [1], who demonstrated that the MPI is a reliable predictor of mortality in patients with peritonitis. Patients with MPI scores above 29 had significantly higher mortality rates, underscoring the need for aggressive management and close monitoring in this high-risk group.

In a study conducted by Qureshi et al., an MPI score of less than 21 had a mortality of 1.9%, a score of 21-29 had 21.9%, and a score of greater than 30 had a mortality of 28.1%. The mortality rate for MPI scores more than 26 was 28.1%, while for scores less than 26 it was 4.3% [15].

Similarly, Kusumoto et al. evaluated the reliability of the MPI in predicting the outcome of patients with peritonitis in 108 patients. A comparison of MPI and mortality showed patients with an MPI score of 26 or less to have a mortality of 3.8%, whereas those with a score exceeding 26 have a mortality of 41.0% [16]. In our study, 13.9% of the patients who succumbed (16.7% of the total cohort) had an MPI score greater than 29.

Notably, as far as postoperative systemic complications were concerned, patients with a higher preoperative MPI were more likely to develop complications like acute kidney injury and pulmonary complications like pneumonia. Acute renal failure was observed in 16.7% of patients, and pulmonary complications in 30.6%, both associated with higher MPI scores, which highlights the severe systemic impact of peritonitis. It is definitely important to monitor renal function in the postoperative period, and proactive respiratory care is essential to reduce pulmonary complications. Li et al. also reported that these complications are more frequent in patients with higher MPI scores, further supporting the index’s role in predicting adverse outcomes [17].

However, our study is limited by its relatively small size. The presence of undiagnosed comorbidities preoperatively may also act as a confounding factor in postoperative outcomes. Additionally, being a single-institute study, it is predisposed to a certain amount of demographic bias. The lower socioeconomic strata of society our hospital commonly caters to may restrict our findings to hospitals with similar characteristics. We therefore propose future multicentric studies with varied demographic groups to further validate our findings.

There are several reasons why this research is important. In the first place, it offers insightful information on the MPI's effectiveness in a particular clinical context, which advances our knowledge of the tool's applicability in forecasting outcomes for perforation peritonitis. Secondly, the research may improve clinical decision-making by verifying the predictive accuracy of the MPI. This would enable more accurate risk classification and customised treatment plans. Third, the results may help guide future studies and possible changes to the MPI to enhance its predictive power even further.

## Conclusions

The findings of this study demonstrate that the MPI is a valuable tool for stratifying patients based on disease severity, with higher scores correlating with worse clinical outcomes, including organ failure, wound complications, and mortality. The demographic analysis revealed a significant male predominance and a relatively young patient population, suggesting regional or demographic factors. The high incidence of organ failure, particularly among those with higher MPI scores, underscores the critical nature of perforation peritonitis and the necessity for intensive care support.
